# Supplementary material for: Design of Artificial Peptide Against HIV-1 Based on the Heptad-Repeat Rules and Membrane-Anchor Strategies
Source: Pharmaceuticals (Basel). 2025 Dec 12;18(12):1881. doi: 10.3390/ph18121881 (PMC12736293; doi:10.3390/ph18121881)
Supplement: Supplementary file 1 [file pharmaceuticals-18-01881-s001.zip › pharmaceuticals-3992183-supplementary.pdf]

Supporting Information

Design of Artificial Peptide Against HIV-1 Based on the Heptad-Repeat Rules and Membrane-Anchor Strategies

Jiali Zhao [1,†], Yan Zhao [1,†], Xiao Qi [1], Xiaojie Lv [1], Yanbai Tang [1], Wei Zhang [1], Qingge Dai [1], Jiaqi Xu [1], Dongmin Zhao [1], Qilu Yan [1], Guodong Liang [1,2,3,*] and Jianping Chen [1,*]

1 Key Laboratory for Candidate medicine Design and Screening Based on Chemical Biology, College of Pharmacy, Inner Mongolia Medical University, Hohhot 010110, China; 13947981607@163.com (J.Z.); zhaoyan@immu.edu.cn (Y.Z.); 20210095@immu.edu.cn (X.Q.); lxj0471@126.com (X.L.); zhangwei0488@163.com (W.Z.); nydaiqingge@163.com (Q.D.); 15648616289@163.com (J.X.); 20250014@immu.edu.cn (D.Z.); 15124832348@163.com (Q.Y.)

2 State Key Laboratory of Natural and Biomimetic Drugs, Peking University, Beijing 100191, China

3 Peptide Drugs Research and Development Center, Zhen-Xiang Technology Co., Ltd., Hohhot 011500, China

* Correspondence: lgd08502214@163.com (G.L.); jianping5817@163.com (J.C.)

† These authors contributed equally to this work.

Content:

1. MALDI-TOF-MS spectrogram of artificial peptides..3
2. CD spectrogram of artificial peptides..7
3. HIV-1 Env-mediated cell-cell fusion cytotoxicity of peptides..10

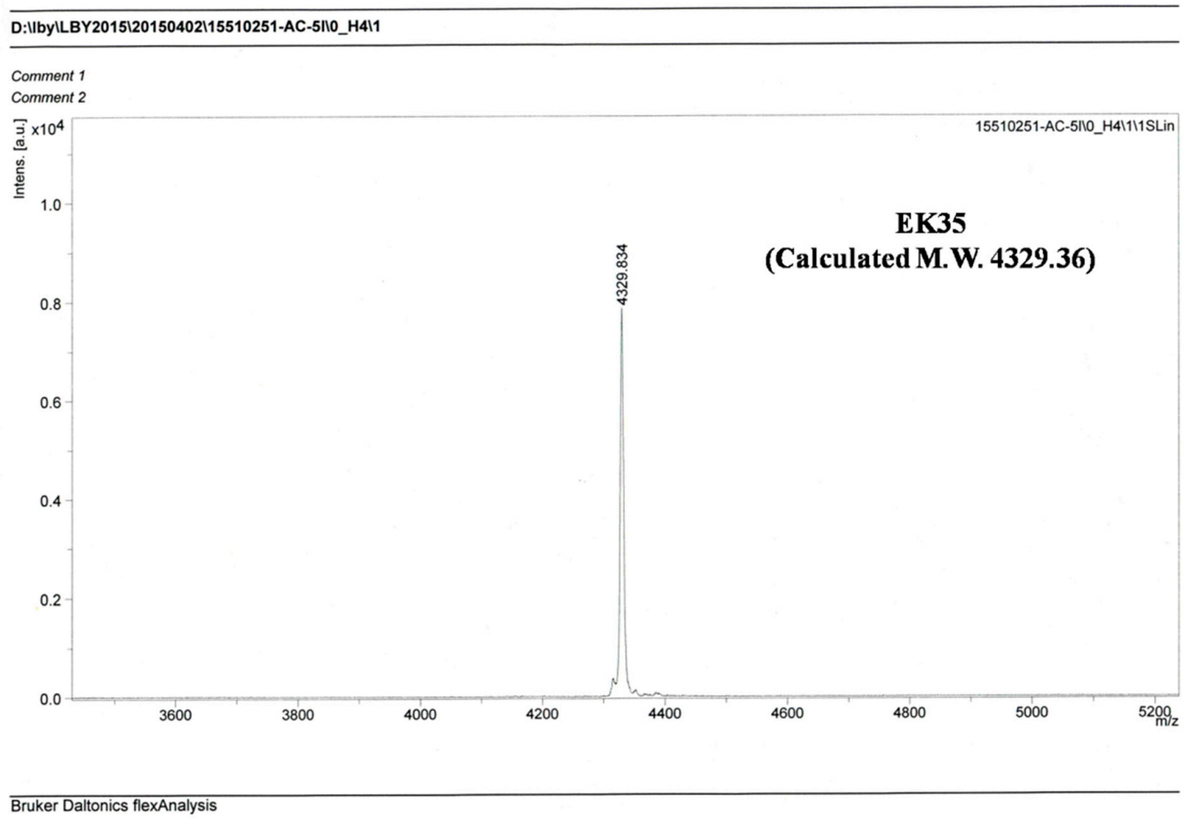

Figure S1. MALDI-TOF-MS spectrogram of EK35.

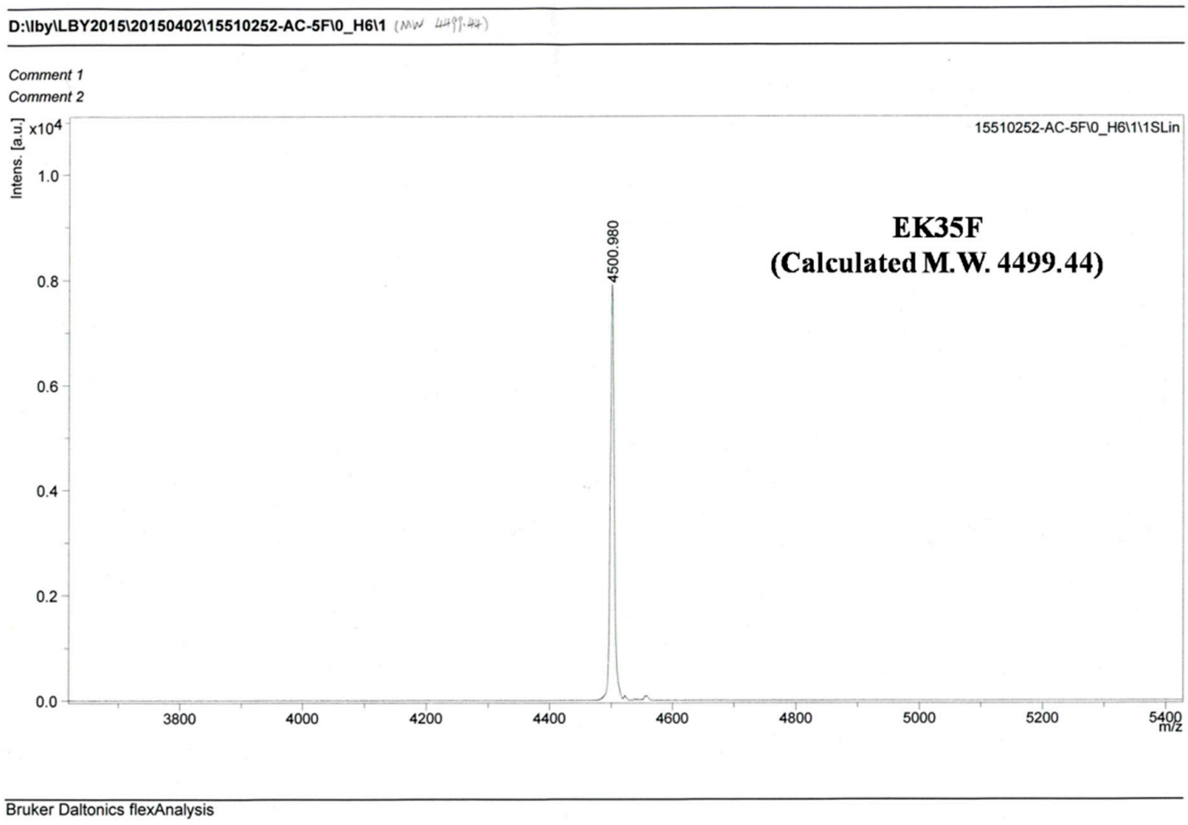

Figure S2. MALDI-TOF-MS spectrogram of EK35F.

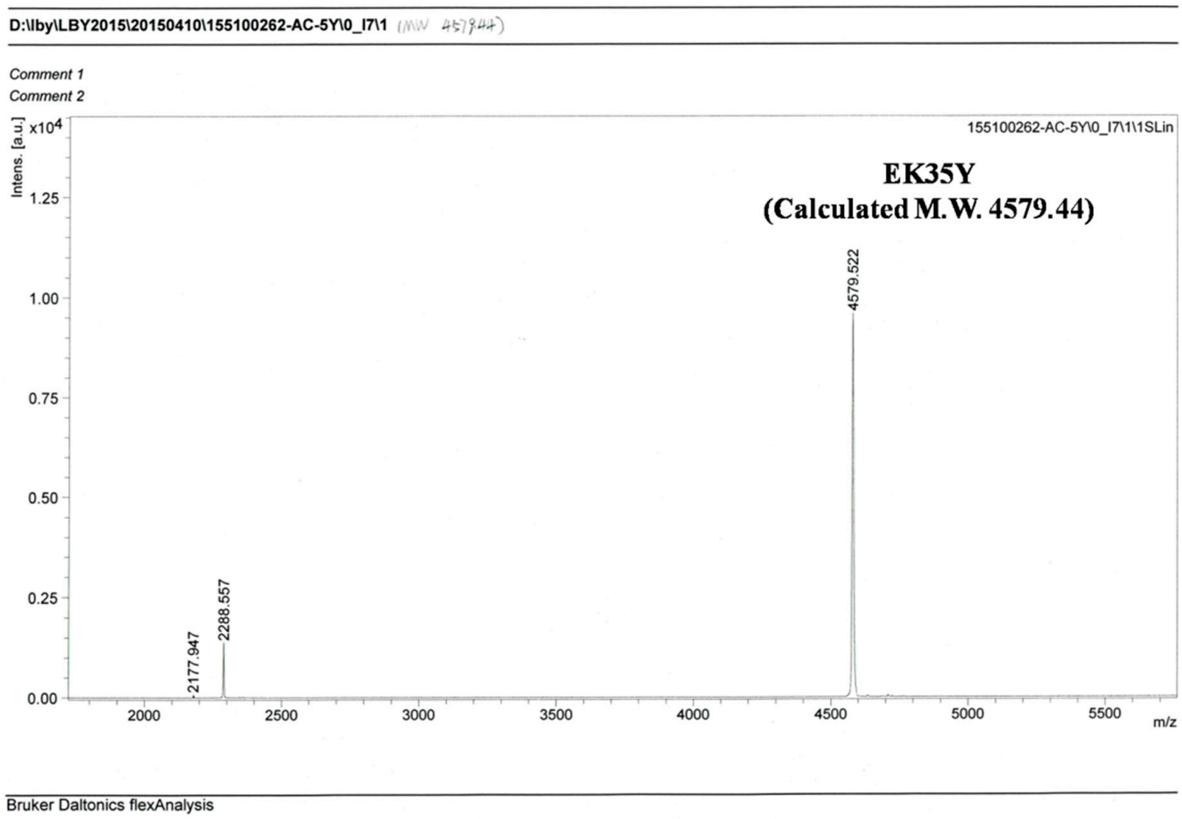

Figure S3. MALDI-TOF-MS spectrogram of EK35Y.

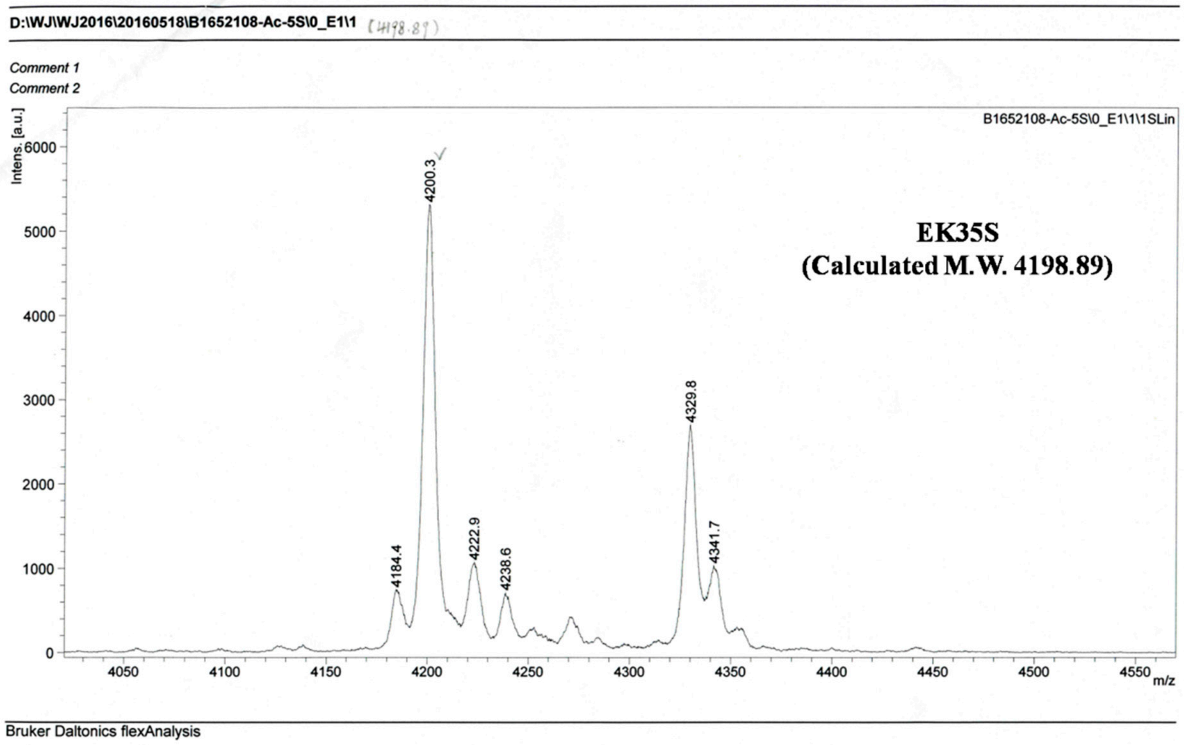

Figure S4. MALDI-TOF-MS spectrogram of EK35S.

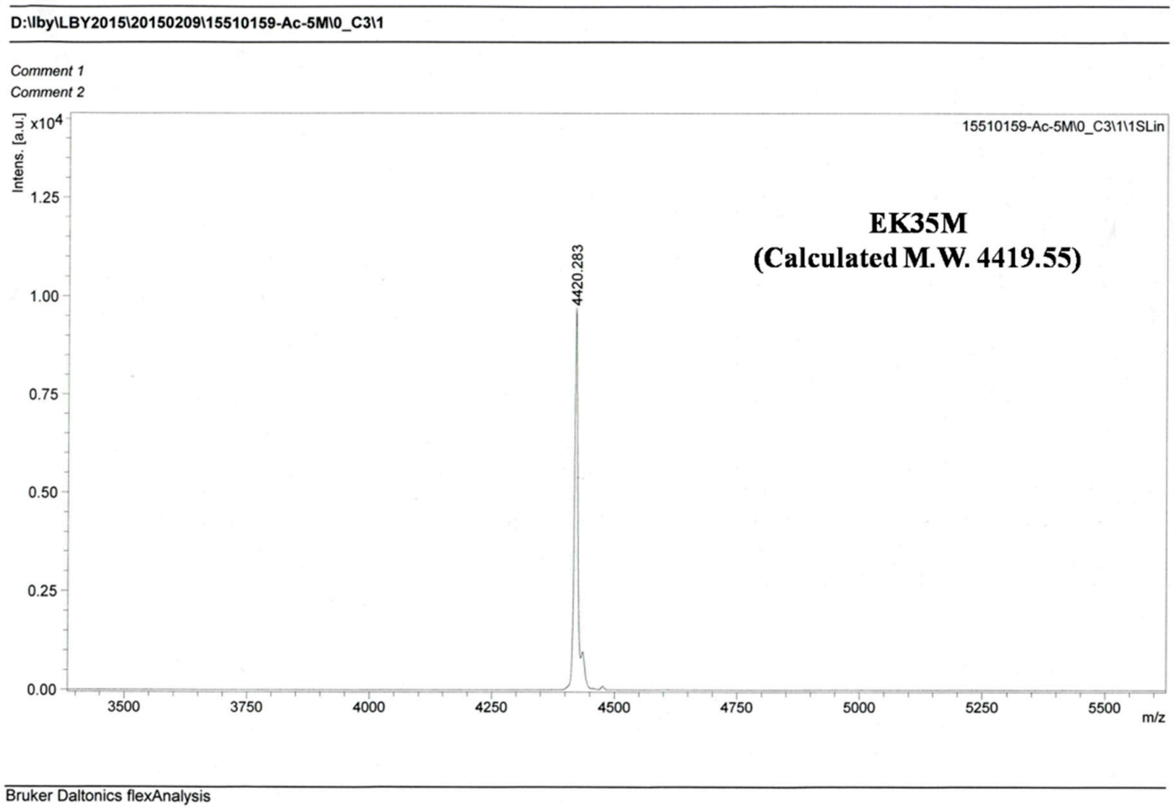

Figure S5. MALDI-TOF-MS spectrogram of EK35M.

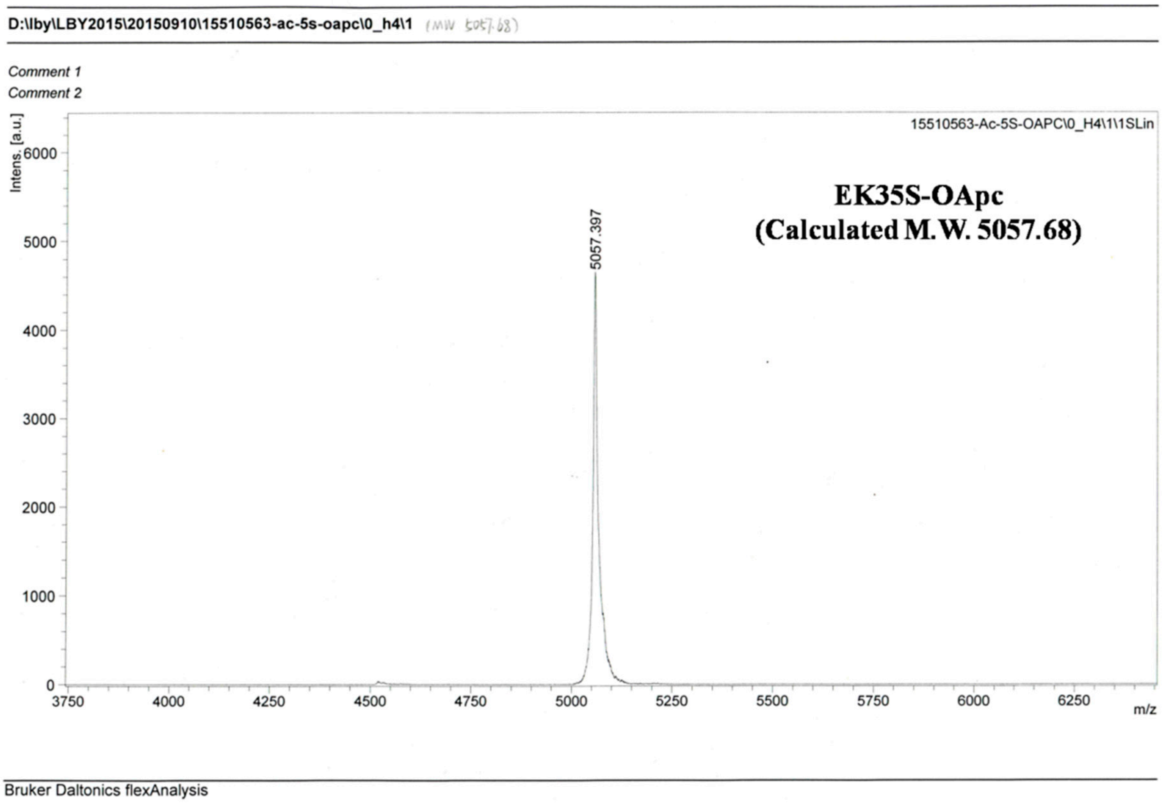

Figure S6. MALDI-TOF-MS spectrogram of EK35S-OApc.

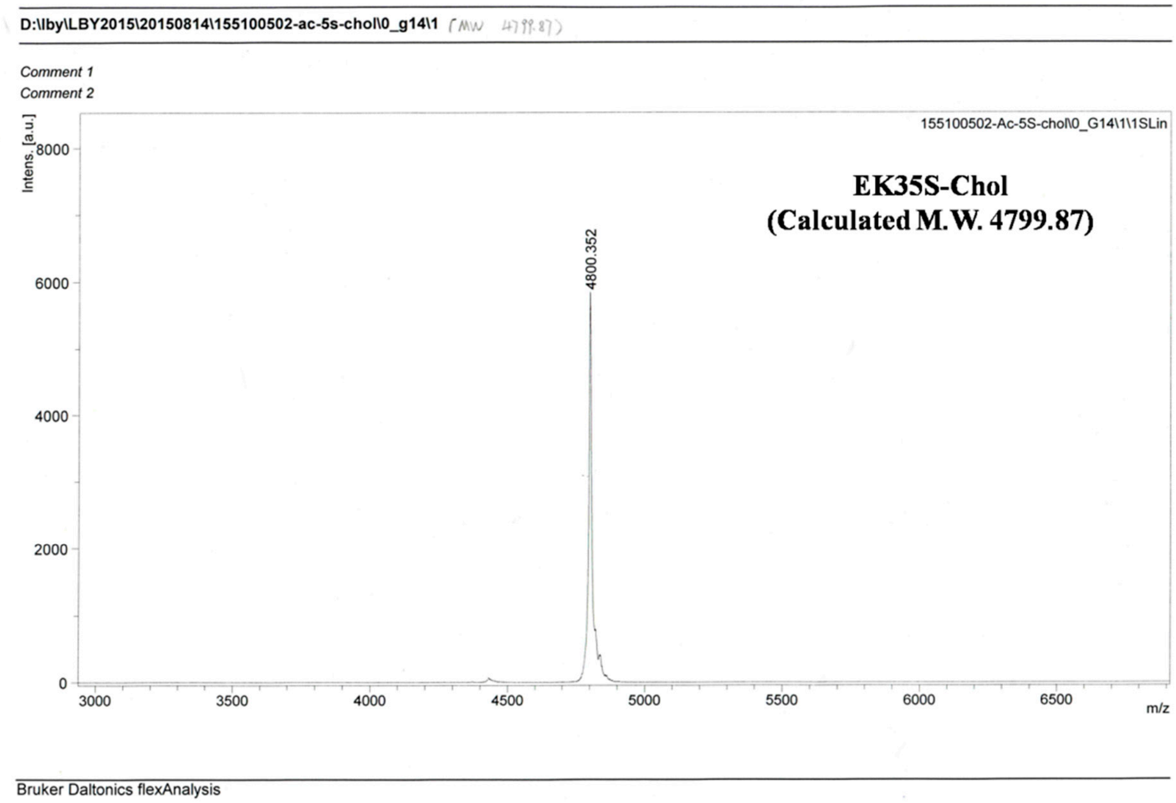

Figure S7. MALDI-TOF-MS spectrogram of EK35S-Chol.

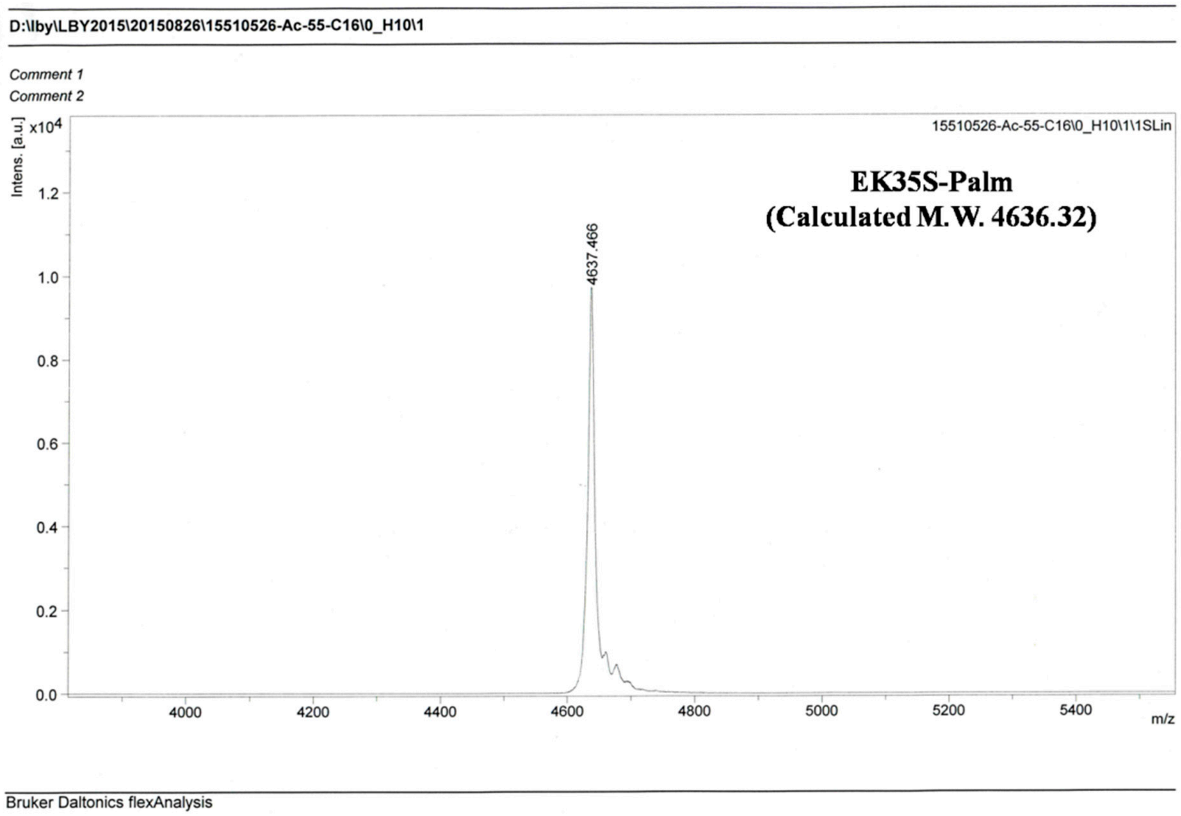

Figure S8. MALDI-TOF-MS spectrogram of EK35S-Plam.

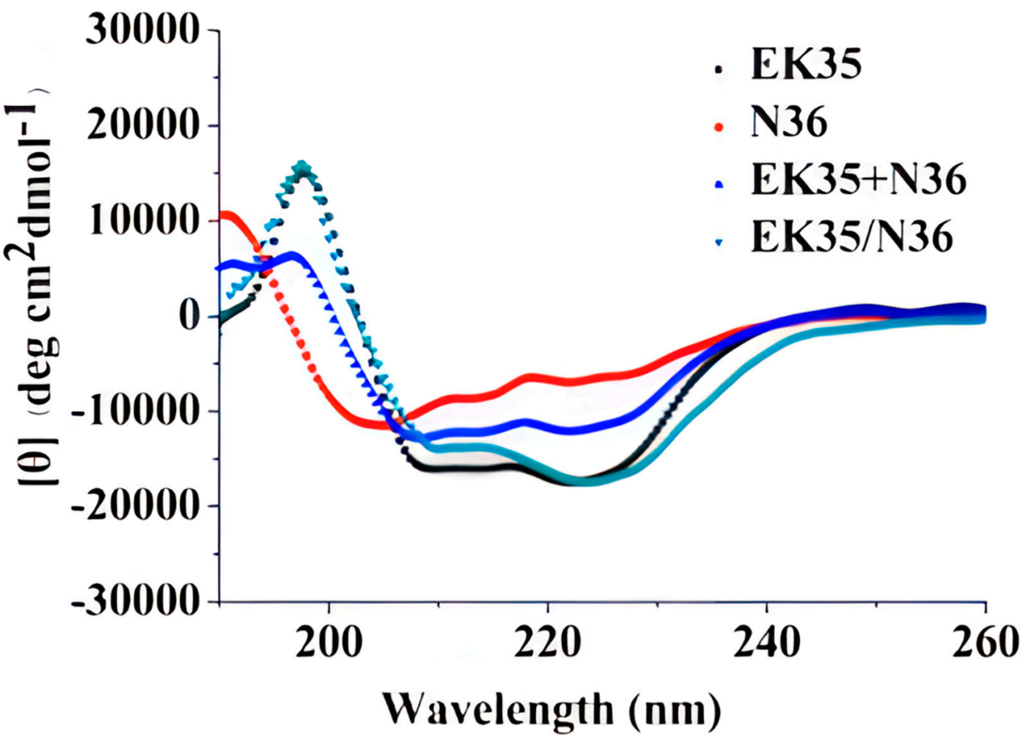

Figure S9. The CD Spectrum of EK35/N36.

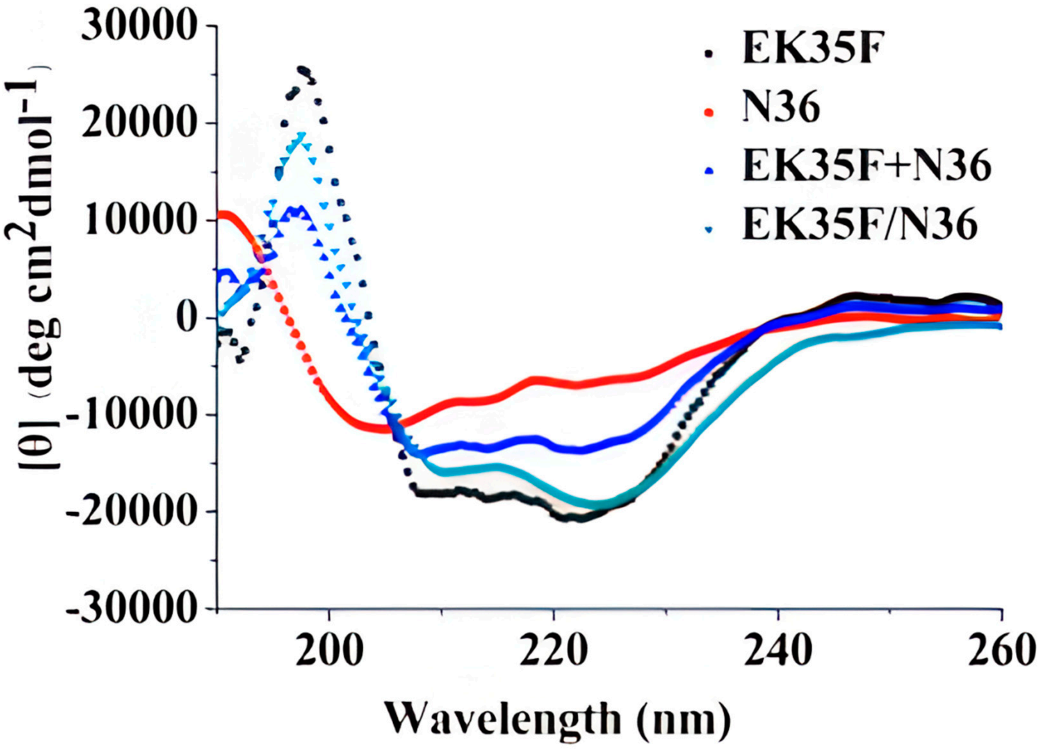

Figure S10. The CD Spectrum of EK35F/N36.

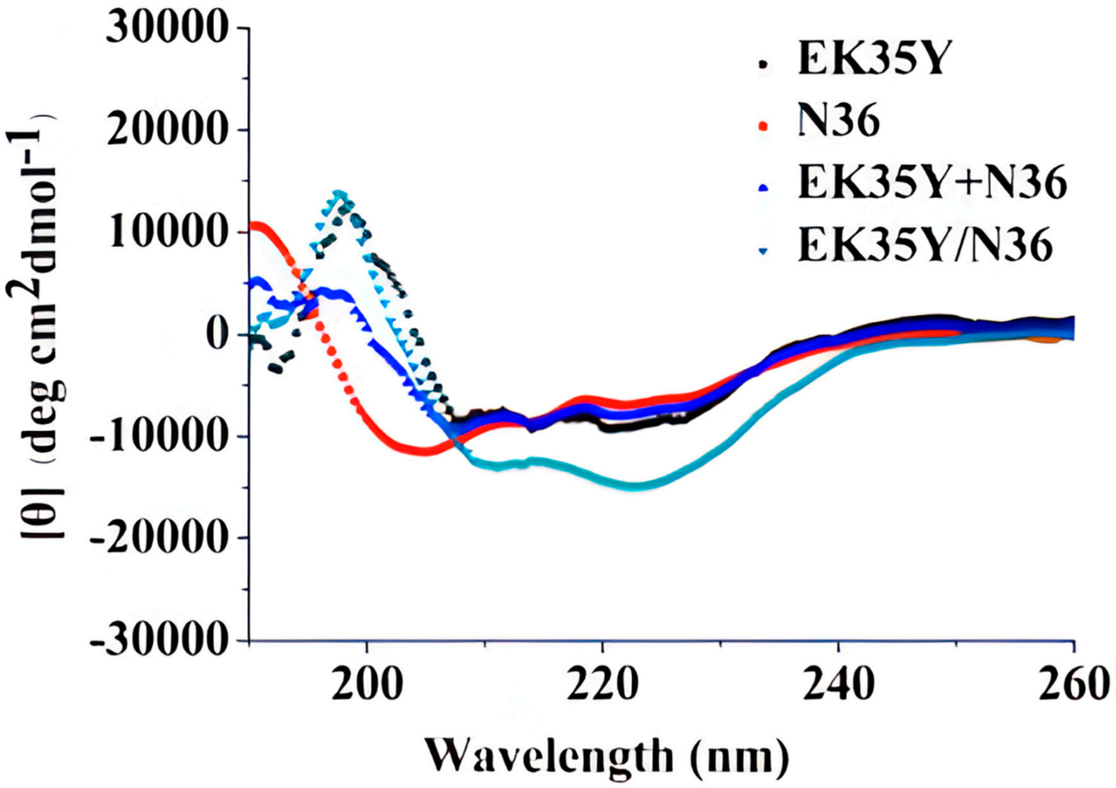

Figure S11. The CD Spectrum of EK35Y/N36.

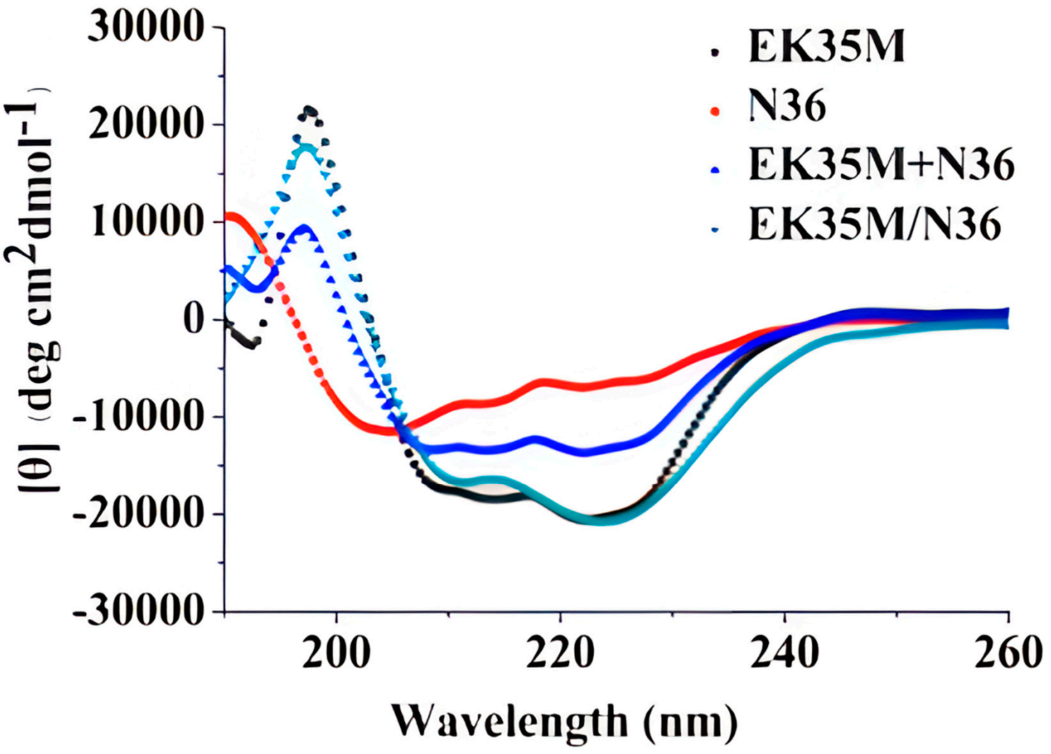

Figure S12. The CD Spectrum of EK35M/N36.

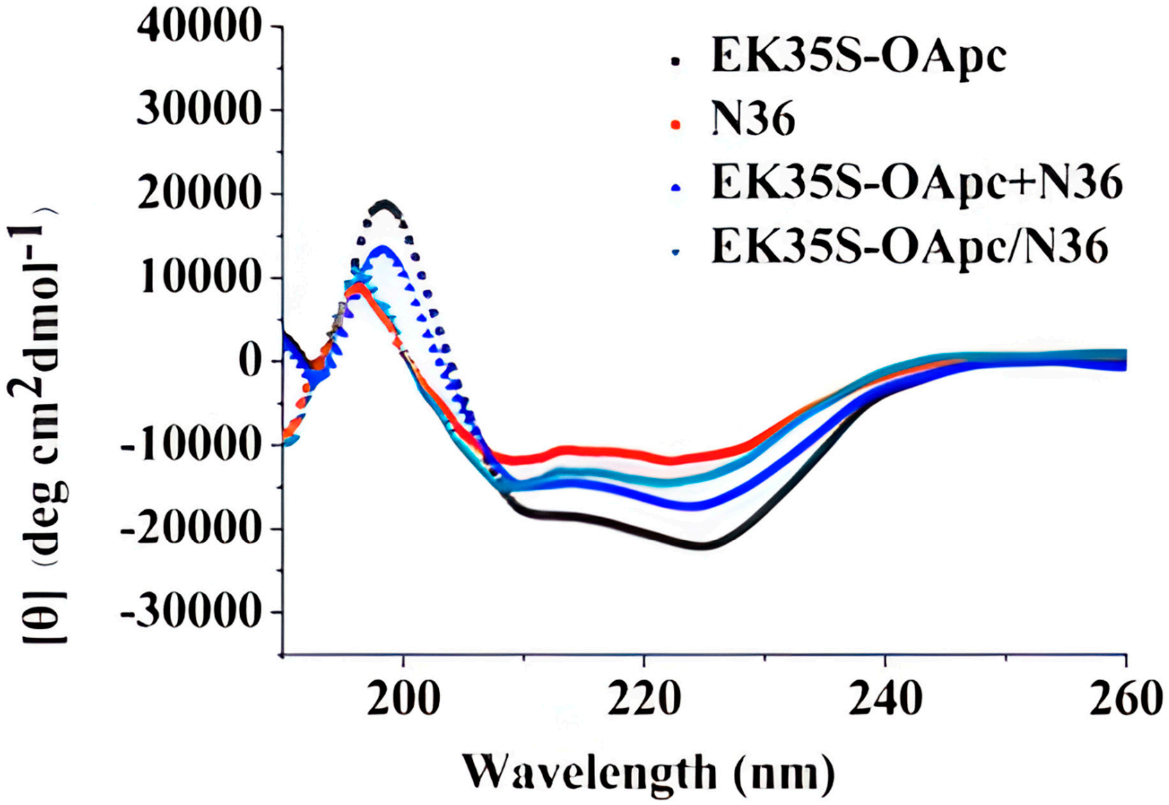

Figure S13. The CD Spectrum of EK35S-OApc/N36.

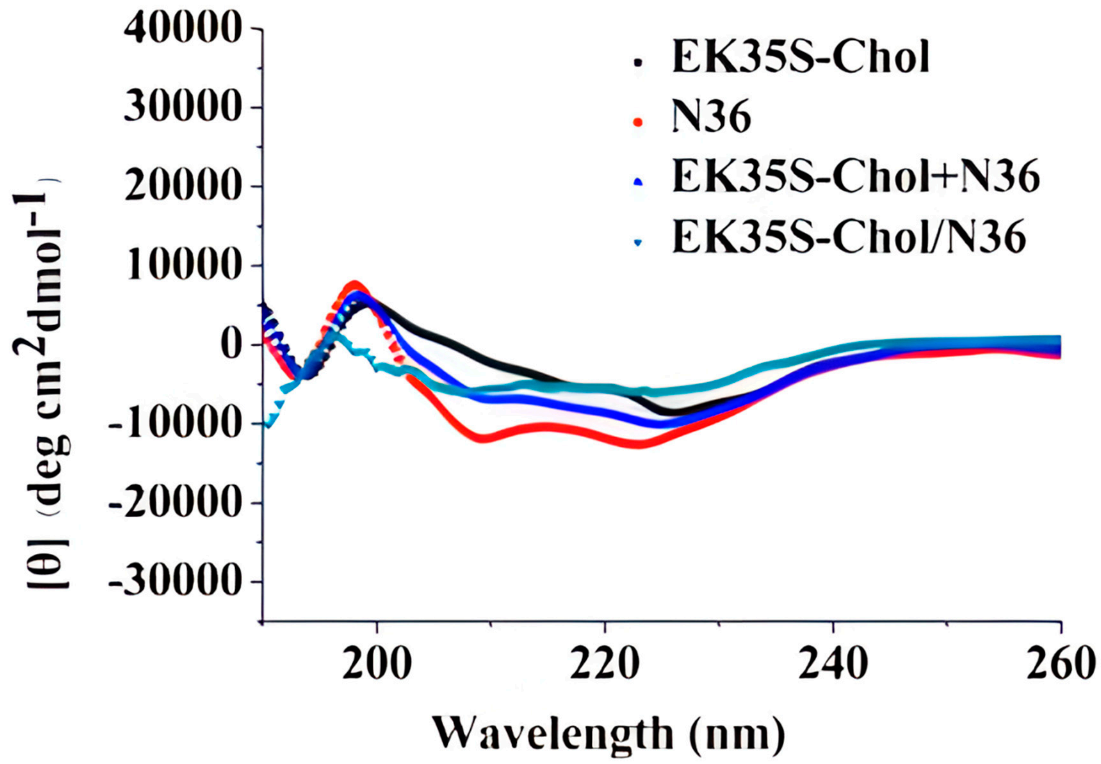

Figure S14. The CD Spectrum of EK35S-Chol/N36.

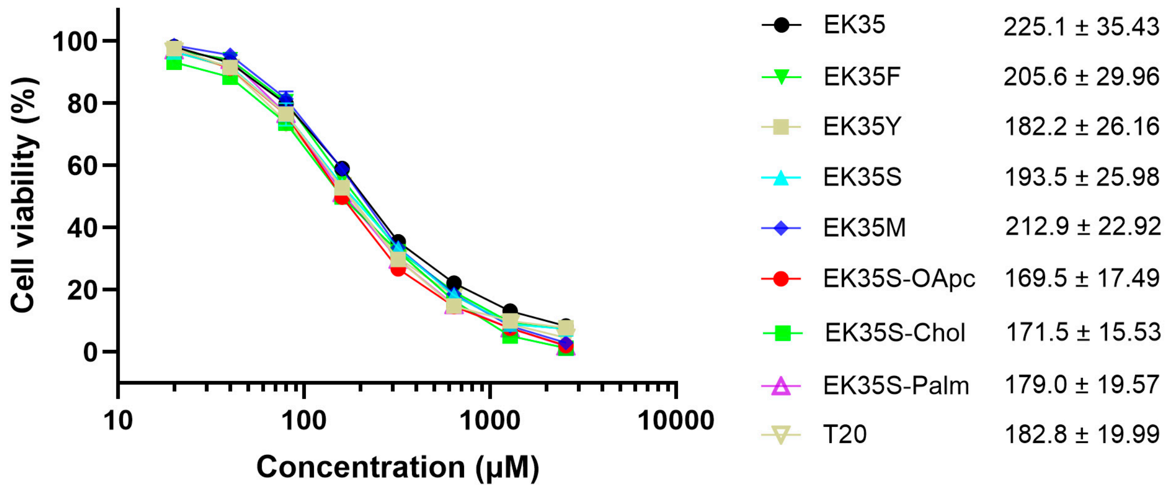

Figure S15. HIV-1 Env-mediated cell-cell fusion cytotoxicity of peptides.
